# Expression analysis of the osteoarthritis genetic susceptibility locus mapping to an intron of the *MCF2L* gene and marked by the polymorphism rs11842874

**DOI:** 10.1186/s12881-015-0254-2

**Published:** 2015-11-19

**Authors:** Colin Shepherd, Andrew J. Skelton, Michael D. Rushton, Louise N. Reynard, John Loughlin

**Affiliations:** Musculoskeletal Research Group, Institute of Cellular Medicine, Newcastle University, 4th Floor Catherine Cookson Building, Framlington Place, Newcastle-upon-Tyne, NE2 4HH UK; Bioinformatics Support Unit, Faculty of Medical Sciences, Newcastle University, 2nd floor, William Leech Building, Framlington Place, Newcastle-upon-Tyne, NE2 4HH UK

**Keywords:** Osteoarthritis, Association, Single nucleotide polymorphism, Gene expression, *MCF2L*, Luciferase reporter assays, Allelic expression

## Abstract

**Background:**

Osteoarthritis (OA) is a painful, debilitating disease characterised by loss of articular cartilage with concurrent changes in other tissues of the synovial joint. Genetic association studies have shown that a number of common variants increase the risk of developing OA. Investigating their activity can uncover novel causal pathways and potentially highlight new treatment targets. One of the reported OA association signals is marked by the single nucleotide polymorphism (SNP) rs11842874 at chromosome 13q34. rs11842874 is positioned within a small linkage disequilibrium (LD) block within intron 4 of *MCF2L*, a gene encoding guanine-nucleotide exchange factor DBS. There are no non-synonymous SNPs that correlate with this association signal and we therefore set out to assess whether its effect on OA susceptibility is mediated by alteration of *MCF2L* expression.

**Methods:**

Nucleic acid was extracted from cartilage, synovial membrane or infrapatellar fat pad tissues from OA patients. Expression of *MCF2L* was measured by quantitative PCR and RNA-sequencing whilst the presence of DBS was studied using immunohistochemistry. The functional effect of SNPs within the 13q34 locus was assessed using public databases and in vitro using luciferase reporter analysis.

**Results:**

*MCF2L* gene and protein expression are detectable in joint tissues, with quantitative differences in the expression of the gene and in the transcript isoforms expressed between the tissues tested. There is an expression quantitative trait locus (eQTL) operating within synovial membrane tissue, with possession of the risk-conferring A allele of rs11842874 correlating with increased *MCF2L* expression. SNPs within the rs11842874 LD block reside within transcriptional regulatory elements and their direct analysis reveals that several show quantitative differences in regulatory activity at the allelic level.

**Conclusions:**

*MCF2L* is subject to a *cis*-acting eQTL in synovial membrane that correlates with the OA association signal. This signal contains several functional SNPs that could account for the susceptibility and which therefore merit further investigation. As far as we are aware, this is the first example of an OA susceptibility locus operating as an eQTL in synovial membrane tissue but not in cartilage.

**Electronic supplementary material:**

The online version of this article (doi:10.1186/s12881-015-0254-2) contains supplementary material, which is available to authorized users.

## Background

Osteoarthritis (OA) is an age-associated disease characterised by the loss of articular cartilage in synovial joints and is multifactorial in its onset. There are currently no pharmacological interventions for patients with OA: total joint replacement is the only effective treatment of end-stage disease but is relatively costly and highly invasive. With the global population increasing in size and in age, and with the incidence of obesity rising, OA is a growing burden on public health budgets. It would be particularly beneficial, therefore, to identify novel therapeutic targets within OA pathways to improve patient outcomes.

While environmental factors can impact upon OA disease progression, it is now clear that genetic factors contribute significantly to the risk of developing OA. Candidate gene and genome-wide association studies (GWAS) have so far identified 16 novel OA risk loci across various joint sites and populations (reviewed in [1] and [2]). These variants are common within the population and have typically small effect sizes that together contribute a significant risk towards developing OA.

During the last decade, it has become apparent that most alleles influencing polygenic traits do so by modulating gene expression, so called expression quantitative trait loci or eQTLs. There are several examples of eQTLs in OA, including those acting on the susceptibility genes *GDF5*, *DIO2* and *ALDH1A2* [3–6].

The majority of OA risk loci do not encompass genes for which there is previously defined mechanistic links to joint development or maintenance. Investigating their role in OA pathology is therefore of significant interest to uncover novel causal pathways and new therapeutic targets. One such risk locus was identified following a GWAS and subsequent meta-analysis of over 19,000 OA cases and 24,500 controls and involved the study of directly typed and imputed single nucleotide polymorphisms (SNPs) [7]. This signal is positioned within *MCF2L* on chromosome 13q34 and is marked by rs11842874. This A/G single nucleotide polymorphism (SNP) has a minor allele frequency (MAF) of 7 % in Europeans and generated an odds ratio (OR) in hip and knee OA of 1.17 for the major, risk-conferring A allele (*p* = 2.1 × 10^−8^).

This signal spans a 13 kb interval of intron 4 of the gene, with rs11842874 having an r^2^ > 0.8 with only six other SNPs within this small linkage disequilibrium (LD) block. *MCF2L* encodes the cytosolic guanine nucleotide exchange factor DBS, known to co-localise and interact with signalling proteins RhoA, Cdc42 and Rac1 [8–15]. These proteins are central to chondrocyte development and hypertrophy whilst Rac1 also mediates the invasive properties of fibroblast-like synoviocytes in rheumatoid arthritis. None of the genes that flank *MCF2L* are stronger OA candidates than this gene. Based on these observations, MCF2L itself is the most likely candidate gene, with none of the surrounding genes having known roles in the musculoskeletal system.

The focus of our study therefore was to characterise *MCF2L* gene and protein expression in joint tissues and to determine whether an eQTL is operating on the gene that correlates with genotype at the association SNP. We also directly assessed the functionality of the seven highly correlated SNPs within the 13 kb LD block.

## Methods

### Patients

Cartilage was obtained from patients undergoing elective total knee or hip replacement surgery due to primary OA. Cartilage was also collected from patients who had undergone a hip replacement due to a neck-of-femur (NOF) fracture. The cartilage of the OA patients had visible lesions and these patients were screened to exclude OA due to trauma or other pathologies. The NOF patients showed no signs or symptoms of hip OA, with the cartilage being macroscopically intact and with no lesions. The cartilage was collected from the tibial plateau and the lateral and medial femoral condyles. For the OA patients, the cartilage was collected at sites distal to the OA lesion. Synovium and infrapatellar fat pad were also collected from knee OA patients. The Newcastle and North Tyneside research ethics committee granted ethical approval for the collection of tissue (REC reference number 09/H0906/72) and verbal and written informed consent for its use and the publication of subsequent data was obtained from each donor. Patients were recruited by The Newcastle upon Tyne Hospitals NHS Foundation Trust, encompassing the Royal Victoria Infirmary and the Freeman Hospital. Patients were recruited by The Newcastle upon Tyne Hospitals NHS Foundation Trust, encompassing the Royal Victoria Infirmary and the Freeman Hospital. Patients were assigned an anonymised Patient ID prior to laboratory members being granted access to their data. We have allocated them another anonymised Patient number to further prevent the identification of individuals that have been included in this study. Details regarding these patients can be found in Additional file 1.

### Nucleic acid extraction

Tissue specimens were snap-frozen at −80 °C and ground to a powder whilst frozen with liquid nitrogen. For cartilage, DNA and RNA were extracted using TRIzol reagent (Life Technologies, UK) as per the manufacturer’s protocol, with the upper aqueous phase separated for RNA isolation, while the interphase and lower organic phase were used to isolate DNA. For synovium and fat pad, DNA and RNA were extracted using the E.Z.N.A. DNA/RNA isolation kit (Omega Biotek, VWR, UK) as per the manufacturer’s instructions.

### Quantitative gene expression analysis

cDNA was synthesised from 1 μg of total RNA using the First Strand synthesis system (Invitrogen, UK) after an initial 30 min treatment with 1 unit of Turbo DNase (Ambion, UK) at 37 °C. Relative gene expression was measured by quantitative PCR using TaqMan® chemistry and reagents. Predesigned TaqMan® assays were used to quantify expression of *MCF2L* (Applied Biosystems, UK) and three housekeeping genes: *HPRT1*, *18S* and *GAPDH* (Integrated DNA Technologies, UK), as described previously [16]. Reactions were performed in triplicate on an ABI PRISM 7900HT Sequence Detection System (Applied Biosystems, UK) and relative *MCF2L* gene expression was calculated by the 2^-ΔCt^ method, where ΔCt is the mean Ct value of the three housekeeping genes subtracted from the Ct value of *MCF2L*. Outliers were removed from the analysis by Grubbs’ test and statistical analysis was performed using the non-parametric Kruskal-Wallis test for analysis between three groups, or the non-parametric Mann–Whitney *U* test for analysis between two groups.

### RNA sequencing (RNA-seq) analysis

For chondrocytes, briefly, total RNA was extracted from the cartilage of 10 hip OA and 6 NOF patients and subjected to 78-base paired-end sequencing using an Illumina Genome Analyser IIx according to the manufacturer’s protocol (these 16 patients are independent of those used in the above quantitative PCR analysis). Sequencing reads were filtered to trim low quality ends before aligning them to known human genome transcript sequences using Salmon, a rapid transcriptome quantification tool and successor to Sailfish [17], which utilized an index based on Ensembl GRCh38 [18]. Bioconductor package cqn was used to normalise read counts and minimise bias due to gene length and GC content. For fibroblast-like synoviocytes and synovial macrophages, publically available RNA-seq raw data in the fastq format was downloaded from the publically available European Nucleotide Archive (ENA; http://www.ebi.ac.uk/ena) for accession numbers SRX105525, SRX105526, SRX544934 and SRX544936. No permissions were required. All samples were QC checked using fastqc (http://www.bioinformatics.babraham.ac.uk/projects/fastqc/) with no trimming. Quantification was performed using Salmon and GRCh38. Fragments per kb of exon per million reads mapped (FPKM) values for transcript variants of the target gene, and an aggregated gene level, were extracted using R (http://www.R-project.org/) and visualized using the ggplot2 library (http://ggplot2.org).

### Immunohistochemistry

Tissue was fixed for 24 h at 4 °C in 4 % paraformaldehyde. Sections were de-waxed in xylene and rehydrated through graded ethanol (100–50 %) and washed in distilled water. Slides were placed into a 2100 Retriever (Aptum Biologics, UK) filled with 10 mM citric acid buffer, pH 6 and processed as per the manufacturer’s protocol. Slides were then rinsed 3 times with deionised water including a final 15 min wash. Sections were washed in TBS (20 mM Tris, pH 7.4 containing 0.9 % NaCl) with agitation for 5 min then incubated in TBS containing 0.3 % hydrogen peroxide to quench endogenous peroxidase activity. Sections were washed in TBS with agitation for 5 min and incubated in 2.5 % horse blocking serum (Vector Laboratories) for 20 min. Excess serum was removed before incubating for 30 min in the presence or absence of an α-DBS antibody (1:350 dilution; Atlas Antibodies, HPA008614). Sections were washed in TBS for 5 min before incubating with ImmPRESS™ (Peroxidase) Polymer Anti-Rabbit Ig Reagent (Vector Laboratories) for 30 min. A further 5 min wash step in TBS was carried out before incubating with 3,3′-diaminobenzidine (Peroxidase (HRP) Substrate Kit, Vector Laboratories) for 5 min. Sections were washed in water for 5 min then counterstained in Mayer’s haematoxylin (Leica Biosystems) for 30 s. Sections were washed in water for 1 min then developed in Scott’s water (20 g/l sodium bicarbonate, 3.5 g/l magnesium sulphate), washed extensively in water for 2 min and dehydrated through ethanol as above, cleared in xylene and mounted in DPX (Sigma Aldrich). Sections were visualised using a DM4000B microscope (Leica).

### Genotyping

Genotyping was performed by pyrosequencing using PyroMark24 (Qiagen, UK) as per the manufacturer’s instructions. The sequence encompassing rs11842874 was PCR amplified using the following primers in a 20 μl reaction volume: biotinylated forward primer 5ʹ-Btn-GACAAAATTCAACGGCAG-3ʹ and reverse primer 5ʹ-TGAGTGACGGCAGCGAGT-3ʹ. Samples were analysed on the PyroMark Q24 MDx platform (Qiagen GmbH, Nordrhein-Westfallen, Germany) using the sequencing primer 5ʹ-GCCCCATCCCGACTC-3ʹ (Sigma-Aldrich) and the PyroMark Gold Q96 reagents Kit, following manufacturer’s instructions. Sequences were generated automatically and an output of allelic ratio produced using PSQ 96 SQA software (Qiagen).

### Online database searches

The Broad Institute [19] online software was used to search for SNPs in high LD (r^2^ > 0.8) with rs11842874 whilst the RegulomeDB [20] online database was used to explore the functionality of the polymorphisms. The GTEx portal [21] online database was used to explore eQTL data (www.gtexportal.org).

### Cloning and luciferase reporter assays

DNA fragments containing each allele of each SNP were amplified by PCR from patient DNA with primers containing restriction sites for MluI (ACGCGT) and XhoI (CTCGAG) (Additional file 2). PCR products and pGL3-promoter vector (Promega) were digested using MluI and XhoI (New England Biolabs) as per the manufacturer’s protocol. Linearised vector and PCR products were then gel purified after agarose electrophoresis using QIAquick gel extraction kit (Qiagen). Ligations were performed using T4 ligase (New England Biolabs) as per the manufacturer’s protocol. Mach1 competent cells (Invitrogen) were used to amplify the ligated products and DNA was purified using PureYield plasmid miniprep or maxiprep systems (Promega). Incorporation of the correct sequence was confirmed by sequencing each allele of each SNP. For the luciferase reporter assay, the human liposarcoma cell line SW872 and the human osteosarcoma cell line U2OS were used. Cells were seeded at 10,000 cells per well in 96 well plates. Twenty four hours later cells were transfected with 50 ng of vector construct as well as 6 ng of a renilla luciferase control vector (pRL-TK, Promega), using FugeneHD transfection reagent (Promega). Cells were incubated for a further 24 h then lysed and luciferase and renilla readings measured using the Dual-luciferase reporter assay system (Promega) as per the manufacturer’s instructions. Values were recorded using a MicroLumat Plus LB96V luminometer (Berthold Technologies UK, Harpenden, UK). A minimum of six independent experiments were performed with six wells transfected per construct and statistical analysis was carried out using the Mann–Whitney *U* test.

## Results

### Characterisation of *MCF2L* gene, transcript and protein expression in primary joint tissues and cells

**Fig. 1:**
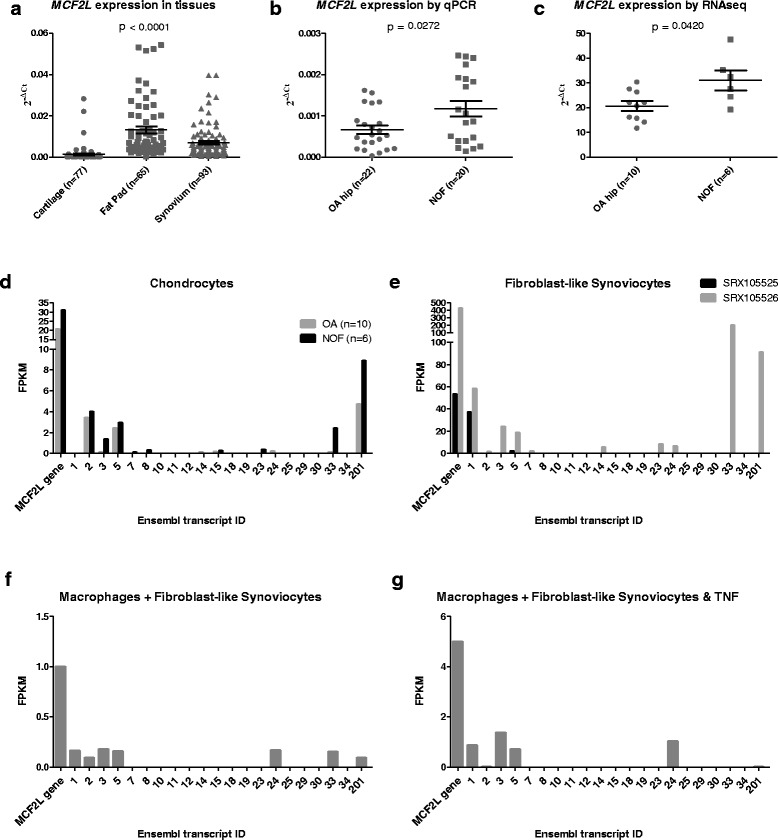
Analysis of relative *MCF2L* expression and of its protein coding transcript isoforms. **a** *MCF2L* expression measured by qPCR in cartilage, synovium and fat pad from OA patient donors. *MCF2L* expression was plotted relative to three housekeeping genes (*GAPDH*, *18S* and *HPRT1*) using the 2^-ΔCt^ method. Each *dot* represents an individual sample. *Lines* represent the mean value of expression and the standard error of the mean. The *p* value was calculated using a non-parametric Kruskal-Wallis test to highlight the differences in mean expression levels between each of the three tissue types tested. **b** *MCF2L* expression measured by qPCR in OA hip and NOF. *Horizontal lines* represent the mean and standard error of the mean. **c** *MCF2L* expression in OA hip and NOF cartilage as measured by RNA-seq. Expression is measured as fragments per kilobase of exon per million fragments mapped (FPKM). *Horizontal lines* represent the mean and standard error of the mean. For b and c, statistical significance was assessed using the Mann–Whitney *U* test and is not corrected for multiple testing. **d** *MCF2L* protein-coding transcript isoform expression as measured by RNA-seq in the OA hip (*grey bars*) and NOF cartilage (*black bars*) samples used in c. **e** Publically available *MCF2L* protein-coding transcript isoform expression as measured by RNA-seq in two fibroblast-like synoviocyte (FLS) samples: SRX105525, shown as *black filled bars*, and SRX105526, shown as *grey filled bars*. **f** Publically available *MCF2L* protein-coding transcript isoform expression as measured by RNA-seq in synovial macrophages co-cultured with FLS. **g** Publically available *MCF2L* protein-coding transcript isoform expression as measured by RNA-seq in synovial macrophages co-cultured with FLS in the presence of tumour necrosis factor (TNF). For **d-g**, the isoforms are labelled with their Ensembl ID on the x-axis and are in the order listed in Additional file 3. Their relative expression, measured as FPKM, is on the y-axis. ‘MCF2L gene’ represents the aggregated expression level of all isoforms

*MCF2L* gene expression has not been described previously in OA-related joint tissues. We therefore initially characterised expression of the gene in three major joint tissues that are readily available to us via joint surgery: cartilage, synovium and fat pad. Quantitative PCR analysis revealed that *MCF2L* expression was highest in fat pad (*n* = 65), followed by synovium (*n* = 93), with comparatively lower levels in cartilage (*n* = 77; hip and knee cartilage samples) (Fig. 1a). A non-parametric Kruskal-Wallis analysis reveals that the differences in expression between these three tissue types are highly significant. We next compared *MCF2L* expression levels between OA and non-OA (NOF) cartilage by quantitative PCR and by RNA-seq analysis. For the qPCR analysis, *MCF2L* expression was found to be 1.7-fold higher in NOF cartilage (Fig. 1b, *p* = 0.027). For the RNA-seq analysis, involving an independent panel of OA hip and NOF patients, *MCF2L* expression was again found to be higher in the NOF patients, this time by 1.5-fold (Fig. 1c, *p* = 0.042).

We further analysed our RNA-seq data to determine which of the 21 *MCF2L* protein coding transcript isoforms predicted by Ensembl (http://www.ensembl.org/index.html; Additional file 3) are expressed in cartilage. We found that five protein coding transcript isoforms were expressed at a fragments per kilobase of transcript per million mapped reads (FPKM) threshold of >1 (Fig. 1d), with no differences in expression between OA and NOF. We also analysed RNA-seq data obtained via the publicly available Sequence Reads Archive (SRA) at NCBI (http://www.ncbi.nlm.nih.gov/sra/) to assess *MCF2L* isoform expression in the two main cell types within healthy synovium tissue: fibroblast-like synoviocytes (FLS) and macrophages. Data from two FLS samples (accession numbers SRX105525 and SRX105526) revealed that ten of the 21 protein-coding transcript isoforms are expressed (FPKM >1) with a high degree of variability in overall gene and isoform expression between the two samples (Fig. 1e). All of the five transcript isoforms expressed in cartilage tissue were also found in one or both of the FLS samples. In synovial macrophages co-cultured with FLS (accession number SRX544934), the FPKM describing overall *MCF2L* gene expression (‘MCF2L gene’) was below 1 thus no isoforms were detected above this level (Fig. 1f). In synovial macrophages co-cultured with FLS in the presence of 20 ng/μl tumour necrosis factor (TNF) (accession number SRX544936), two transcript isoforms were expressed at FPKM >1 (Fig. 1g).

In summary, we note that between chondrocytes, synovial macrophages and synovial fibroblasts, a total of 11 transcript isoforms were expressed. Isoform *MCF2L*-003 was the only one expressed in chondrocytes, fibroblast-like synoviocytes and macrophages co-cultured with FLS and TNF; the remaining ten isoforms were expressed in a maximum of two of the three cell types tested. We were unable to directly compare overall gene or isoform expression between cell types as these were separate experiments. The conclusions drawn from these data should be interpreted with caution as the n number used for the synovial fibroblasts and macrophage data are extremely low. We were unable to analyse adipocyte RNA-seq data due to a lack of suitable publically available data.

In addition to these analyses, we performed immunohistochemistry on cartilage, synovium and fat pad from OA patients with an α-DBS (the protein product of *MCF2L*) primary antibody, and compared staining to that found in tonsil tissue as a positive control (Additional file 4). DBS protein was detectable in chondrocytes, synovial fibroblasts, synovial macrophages and adipocytes. Comparatively dark staining was seen around the plasma membrane and within the cytosol in tonsil lymphocytes. Staining was less intense in the joint tissue cells and was more evenly distributed throughout the cytosol, with less concentrated staining around the plasma membrane.

In summary, *MCF2L* is expressed in the three joint tissues that we analysed but with quantitative differences between the tissues and in the relative abundance of *MCF2L* transcript isoforms.

### Quantitative *MCF2L* expression in cartilage, synovium and fat pad stratified by genotype at the OA-risk locus rs11842874

Having shown that *MCF2L* is expressed at the gene and protein level in the joint tissues, we next assessed whether an eQTL was operating at the rs11842874 locus in one or more of these tissues. Having measured *MCF2L* expression in OA cartilage, fat pad and synovium tissue (Fig. 1a), we then genotyped the same individuals at rs11842874 and stratified *MCF2L* expression by genotype at this SNP. Since the MAF of rs11842874 is low (7 % in Europeans), major allele homozygotes (AA) were compared to minor allele carriers (AG and GG combined) to ensure sufficient power in our statistical analyses. Of the 77 cartilage samples analysed in Fig. 1a, 75 were genotyped whilst all 65 of the fat pad samples and all 93 of the synovium samples were genotyped.

**Fig. 2:**
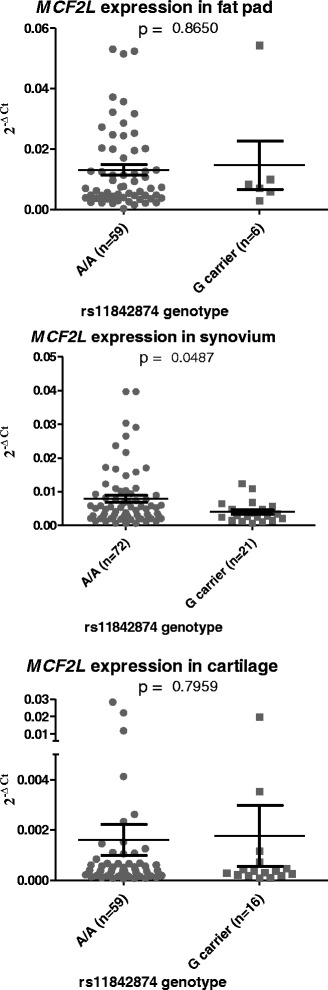
eQTL analysis of *MCF2L* in joint tissues. The individuals included in the expression analysis of Fig. 1a were genotyped at rs11842874. Gene expression, as measured by qPCR, was then stratified by genotype at the SNP, with individuals placed into two genotypic groups: AA homozygotes and G carriers (AG plus GG). The data is presented as columnar scatter plots for each tissue type. Each data point represents the mean of three technical repeats. *Horizontal lines* represent the mean and standard error of the mean. Statistical significance was assessed using the Mann–Whitney *U* test and is not corrected for multiple testing

In cartilage and fat pad, we found no significant difference in *MCF2L* gene expression between the two genotypes (Fig. 2, *p* > 0.05). In synovium tissue however, *MCF2L* expression was significantly higher in major allele homozygotes (*p* = 0.0487). We stratified gene expression by sex and joint for each tissue type but saw no sex-specific or joint-specific correlations (data not shown). There is evidence, therefore, of an eQTL at this locus that correlates with the OA-association signal, with possession of the risk-conferring A allele correlating with increased *MCF2L* expression in OA synovium tissue.

In addition to our own eQTL analysis, we carried out a search of the GTEx project online database. This database contains data relating to tissue specific eQTLs described in a variety of tissue types. Unfortunately, for the purpose of this study, the project does not currently contain data relating to our tissue types of interest. Nevertheless, *MCF2L* is subject to an eQTL in skeletal muscle (*p* < 0.003) and oesophagus muscularis (*p* < 0.0003). However, the regulatory SNPs driving this eQTL are in a region of *MCF2L* that has low LD (r^2^ ≤ 0.1) with rs11842874 (Additional file 5).

Due to the low MAF of rs11842874 and the absence of *MCF2L* transcript SNPs in LD with this intronic SNP, which precluded our identification of a testable number of compound heterozygotes, we were unable to directly test allelic expression of *MCF2L* by allelic expression imbalance (AEI) analysis (also known as differential allelic expression (DAE); [4, 16]). Furthermore, we were unable to successfully design reliable assays for the limited number of suitable *MCF2L* transcript SNPs that exist for this gene.

### The rs11842874 LD block is functional

**Fig. 3:**
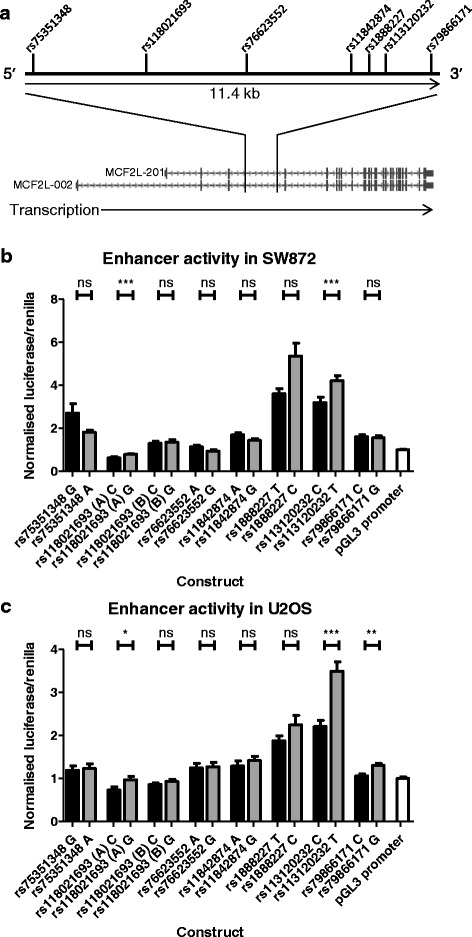
Functional analysis of SNPs within the rs11842874 linkage disequilibrium block. (**a**) Schematic diagram of the 7 SNPs within the LD block. The SNPs are within an 11.4 kb region located within the *MCF2L* gene body (intron 3 relative to transcript isoforms 002 and 201); adapted from the UCSC genome browser. Luciferase reporter analyses were performed in (**b**) SW872 liposarcoma and (**c**) U2OS osteosarcoma cells. Cells were transfected with luciferase reporter constructs and a renilla reporter vector. Luciferase activity was quantified and normalised to renilla activity and to the empty luciferase vector. Mean luciferase/renilla data is plotted, with *error bars* representing standard error of the mean from six technical repeats and a minimum of six biological repeats. SNPs are presented in their physical order on the x-axis. Major (risk) alleles are presented as *black filled bars*, minor alleles as *grey filled bars*. Two fragments encompassing rs118021693 were analysed; fragment A and the larger fragment B. Statistical significance represents the difference between alleles for each SNP and was assessed using the Mann–Whitney *U* test, and is not corrected for multiple testing. *ns* not-significant, *p < 0.05, **p < 0.01, ***p < 0.001

The OA risk locus is narrow and contains rs11842874 and only six SNPs that have an r^2^ > 0.8 with this index SNP (Table 1); all reside within intron 3 of *MCF2L* with reference to transcript isoforms MCF2L-002 and MCF2L-201 (Fig. 3a). An analysis of online public databases revealed that this region is predicted to have transcriptional regulatory activity, as summarised in Additional file 6. Each of the 7 SNPs resides within a domain correlating with either weak transcriptional activity or strong enhancer effects.

**Table 1:** Six SNPs in high (r^2^ > 0.8) linkage disequilibrium with rs11842874

			Pairwise linkage disequilibrium with rs11842874	
SNP	Alleles (major/minor)	Minor allele frequency	r2	D’
rs75351348	G/A	0.085	0.85	1
rs118021693	C/G	0.058	1	1
rs76623552	A/G	0.058	1	1
rs11842874	A/G	0.066	–	–
rs1888227	T/C	0.050	1	1
rs113120232	C/T	0.067	0.867	1
rs79866171	C/G	0.058	1	1

For each SNP, the major and minor alleles, minor allele frequencies, and pairwise r^2^ and D’ values are listed for the HapMap Central European population

We used a luciferase dual-reporter assay system to directly investigate the functionality of each allele of each of the seven SNPs. DNA surrounding each SNP was cloned separately into a luciferase reporter construct and constructs were created for both alleles. For rs118021693, one region of 772 bp (rs118021693 (A)) was cloned and one region of 1216 bp (rs118021693 (B)) was cloned; the larger insert incorporated a region with predicted insulator activity. To select an appropriate human cell line for transfection, we tested a variety of lines for expression of *MCF2L* (data not shown). Most did not express the gene (this included transformed synovial fibroblasts) or expressed it at a low level. We settled on two cells lines that did express the gene and which would therefore be synthesising transcriptional proteins regulating *MCF2L* expression: the liposarcoma cell line SW872 and the osteosarcoma cell line U2OS. Both are of mesenchymal stem cell origin and as such are developmentally related to the primary joint tissues that have been studied in this report.

We observed significant allelic differences (*p* < 0.05) for two of the seven SNPs in SW872 cells (rs118021693 and rs113120232; Fig. 3b) and for three of the seven SNPs in U2OS cells (rs118021693, rs113120232 and rs79866171; Fig. 3c). Two of these SNPs are common between the two cell lines: rs118021693 and rs113120232. As noted above, we analysed two regions encompassing rs118021693 and it was the smaller of the two (the “A” region) that showed an allelic effect in SW872 and U2OS. Both allelic forms of rs118021693 showed reduced luciferase activity relative to the control plasmid (pGL3 promoter, far right bar in Fig. 3b and c), and in both cell lines the major C allele showed a greater reduction in luciferase activity relative to the minor G allele. Both allelic forms of the rs113120232 region showed increased luciferase activity relative to the control plasmid, and this effect was greater for the minor T allele relative to the major C allele in both cell lines.

In summary, this luciferase analysis demonstrated: 1) that the region of intron 4 of *MCF2L* encompassing the OA association signal has transcriptional regulatory activity; 2) that three of the seven SNPs correlating with the signal show statistically significant differential allelic expression and; 3) that for two of these three SNPs, the effects are observed in both cell types examined and in the same allelic direction.

## Discussion

We initially demonstrated the expression of *MCF2L* and the presence of its protein product, DBS, in three major tissues of the synovial joint; cartilage, synovial membrane and infrapatellar fat pad. This analysis also highlighted quantitative differences in the expression of the gene between these tissues, as well as differences in relative transcript isoform expression. We subsequently identified a synovium tissue eQTL that operates on *MCF2L* and which correlates with the 13 kb association signal marked by SNP rs11842874. Finally, we uncovered allelic functionality at SNPs within the association interval, which is located within intron 4 of the gene. Our study therefore provides a potential mechanism through which the rs11842874 signal acts as an OA susceptibility locus, namely the modulation of *MCF2L* expression in synovium tissue.

DBS is a guanine nucleotide exchange factor that has not been investigated to a great degree. However, and as noted in the Introduction, it is known to interact with signalling proteins involved in chondrocyte development and hypertrophy, and with proteins involved in the invasive properties of fibroblast-like synoviocytes [8–15]. It does therefore have a known role in joint tissue biology. In our RNA-seq analysis we noted that *MCF2L* expression was higher in synovial macrophages in the presence of TNF than in its absence. TNF and other proinflammatory cytokines have a well-defined role in cartilage degradation and OA synovitis [22] and this result may therefore indicate that *MCF2L* is involved in this inflammatory pathway in synovial macrophages.

Our discovery of an eQTL acting in synovium tissue but apparently not in cartilage emphasises that OA is not exclusively a cartilage disease but instead has joint-wide effects. We have previously reported on synovium tissue eQTLs in OA patients [16, 23, 24], but as far as we are aware, this is the first example of one operating in synovium tissue but not in cartilage. It is of course possible that the eQTL is active in cartilage and that we lacked the power to detect it, despite analysing the cartilage of over 75 OA patients.

In the synovium tissue eQTL analysis, increased expression of *MCF2L* correlated with the risk-conferring A allele of rs11842874. This is the common allele of the SNP. In the luciferase studies, the two SNPs that demonstrated a consistent effect in both SW872 and U2OS cells, rs118021693 and rs113120232, each showed reduced expression of their common alleles relative to their rare alleles. Due to the very high LD in the association interval, the common alleles of rs118021693 and rs113120232 are on the same haplotype as the common allele of rs11842874. There is therefore a contradiction between the results of the eQTL and the luciferase studies. This can be accounted for by a number of differences between the two experimental approaches. For example: 1) the luciferase assays used liposarcoma and osteosarcoma cells whereas the eQTL was observed in synovial cells; 2) the luciferase assays used transformed cells whereas the eQTL analysis used tissue and; 3) in a luciferase assay, a linearised piece of DNA is studied, which cannot realistically mimic the chromatin interactions present in the cell in vivo. The purpose of the luciferase assay was to assess whether the two alleles of a SNP mediate differential gene expression; if they do, this implies that the SNP has the capacity to act as an eQTL. The luciferase assay is therefore an in vitro test of SNP functionality whereas the tissue-based eQTL analysis measured the actual activity of the *MCF2L* gene in the synovium tissue of patients at the time of their operation and according to their rs11842874 genotype.

Overall, we have described tissue-specific effects of genotype at rs11842874 on *MCF2L* expression, which is in agreement with recent studies suggesting that many genetic variants associated with complex traits are likely to affect gene expression through eQTLs in a tissue specific manner [25]. Further functional analyses of the SNPs within the association interval, of *MCF2L* and of DBS are now justified to fully comprehend how this 13q34 locus increases OA susceptibility.

## Conclusion

*MCF2L* is subject to a *cis*-acting eQTL in the synovial membrane tissue of patients that correlates with the OA association signal at this locus. This may therefore be the mechanism through which this genetic susceptibility is acting. Furthermore, there are predicted and experimentally confirmed functional SNPs within the association interval that correlate with the signal and which show differential allelic expression. These now merit further investigation as potential drivers of the association signal.

### Availability of data and materials

Data sets supporting the results of this article are available in the LabArchives repository, under DOI: 10.6070/H45M63Q4. The raw data supporting the findings presented here are also available from the corresponding author upon request.

## Additional files

Additional file 1:
**Details of the patients studied in this report for A) qPCR analysis and B) RNA sequencing analysis.** (XLSX 22 kb) Additional file 2:
**The 21** ***MCF2L*** **protein coding transcript isoforms listed by Ensembl and analysed as part of Fig.** 1 **.** (PDF 293 kb) Additional file 3:
**Primers used for the cloning of the seven SNPs within the association interval for their analysis by luciferase expression.** (PDF 273 kb) Additional file 4:
**DBS protein expression in joint tissues from OA patients.** Immunohistochemistry staining of fixed and wax embedded synovium, fat pad and cartilage tissue, with healthy tonsil tissue used as a positive control. Tissue was stained with an anti-DBS primary antibody (α-DBS). Also shown is the no primary antibody (NPA) control for each tissue. Bars = 100 μm (tonsil, synovium, fat pad) or 200 μm (cartilage). (PDF 371 kb) Additional file 5:
**An eQTL that regulates** ***MCF2L*** **gene expression in skeletal muscle and oesophagus muscularis.** A search of the GTEx website identified an *MCF2L* eQTL operating in skeletal muscle and oesophagus muscularis. The eQTL is located in a 7 kb LD block containing ten SNPs in high LD (r^2^ > 0.8). It is approximately 42 kb downstream of the rs11842874 LD block and is in low LD with this block. The table lists the ten SNPs and their r^2^ and D’ values relative to rs11842874. HG19 coordinates refer to human genome version 19. (PDF 217 kb) Additional file 6:
**Bioinformatics database search of the SNPs in high LD with rs11842874.** Detailed are the chromatin states around the SNPs in several types of joint-related cell from various origins including osteoblasts, mesenchymal stem cells, differentiated chondrocytes and adipocytes; characterising the regulatory properties of the region. (PDF 213 kb)

## Abbreviations

AEI: allelic expression imbalance
eQTL: expression quantitative trait locus
FLS: fibroblast-like synoviocyte
FPKM: fragments per kilobase of transcript per million reads mapped
GWAS: genome-wide association scan
LD: linkage disequilibrium
MAF: minor allele frequency
NOF: neck of femur fracture
OA: Osteoarthritis
OR: odds ratio
SNP: single nucleotide polymorphism
SRA: sequence reads archive
TNF: tumour necrosis factor
