# Supplementary material for: Expression analysis of the osteoarthritis genetic susceptibility locus mapping to an intron of the MCF2L gene and marked by the polymorphism rs11842874
Source: BMC Med Genet. 2015 Nov 19;16:108. doi: 10.1186/s12881-015-0254-2 (PMC4653905; doi:10.1186/s12881-015-0254-2)
Supplement: Additional file 2: — The 21 MCF2L protein coding transcript isoforms listed by Ensembl and analysed as part of Fig. 1 . (PDF 293 kb) [file 12881_2015_254_MOESM2_ESM.pdf]

Additional File 2. Primers used to clone SNPs in LD region for luciferase reporter assay, cloning sites for restriction enzymes MluI (ACGCGT) and XhoI (CTCGAG) underlined and cloned fragment sizes. Primers were designed to include the SNP of interest within the middle of the fragment.

SNP	Forward Primer (5' - 3')	Reverse Primer (5' - 3')	Cloned fragment size including primer sequence (bp)
rs75351348	GGGGACGCGTGTGGGAAAACAATGTGAGAAAGA	GGGGCTCGAGAGAAAACACAGCTCCTCAGAAAA	548
rs118021693 (A)	GGGGACGCGTGAGATGCGTCTGGCTCTGTC	GGGGCTCGAGGTCGGTTCTGAGCTGTGTCTG	772
rs118021693 (B)	GGGGACGCGTGAGATGCGTCTGGCTCTGTC	GGGGCTCGAGAGACACCCAGAGCTGCACAT	1216
rs76623552	GGGGACGCGTATGAATTCCTAGGCCTCACTCTC	GGGGCTCGAGGGCTCTGAACCAGTTTAGTCTGA	609
rs11842874	GGGGACGCGTCCAACAGGAGTGTGTATTGTGTG	GGGGCTCGAGACCCAAGATGAGAAGAGGAGTCT	634
rs1888227	GGGGACGCGTTCGTTGGTAACTGGTTTCTCATT	GGGGCTCGAGTTGGGGTAGAATAACTCAAGCTG	583
rs113120232	GGGGACGCGTGCTGCCAGCTTGAGTTATTCTAC	GGGGCTCGAGTGTCTCCTCTCCTACCACATTTC	587
rs79866171	GGGGACGCGTTCGTTGGTAACTGGTTTCTCATT	GGGGCTCGAGCCATCTCTGCTTCCTTTTCTGTA	703
