# Supplementary material for: Expression analysis of the osteoarthritis genetic susceptibility locus mapping to an intron of the MCF2L gene and marked by the polymorphism rs11842874
Source: BMC Med Genet. 2015 Nov 19;16:108. doi: 10.1186/s12881-015-0254-2 (PMC4653905; doi:10.1186/s12881-015-0254-2)
Supplement: Additional file 3: — Primers used for the cloning of the seven SNPs within the association interval for their analysis by luciferase expression. (PDF 273 kb) [file 12881_2015_254_MOESM3_ESM.pdf]

Additional File 3. Ensembl *MCF2L* transcript variant names and Ensembl ID.

Ensembl transcript ID	Ensembl transcript name
MCF2L-001	ENST00000375608
MCF2L-002	ENST00000397030
MCF2L-003	ENST00000375597
MCF2L-005	ENST00000535094
MCF2L-007	ENST00000397021
MCF2L-008	ENST00000473345
MCF2L-010	ENST00000420013
MCF2L-011	ENST00000413354
MCF2L-012	ENST00000439475
MCF2L-014	ENST00000441756
MCF2L-015	ENST00000397036
MCF2L-018	ENST00000397024
MCF2L-019	ENST00000486806
MCF2L-023	ENST00000423251
MCF2L-024	ENST00000421756
MCF2L-025	ENST00000433807
MCF2L-029	ENST00000453297
MCF2L-030	ENST00000409954
MCF2L-033	ENST00000397017
MCF2L-034	ENST00000261963
MCF2L-201	ENST00000375604
