# Supplementary figures and images for: Expression analysis of the osteoarthritis genetic susceptibility locus mapping to an intron of the MCF2L gene and marked by the polymorphism rs11842874
Source: BMC Med Genet. 2015 Nov 19;16:108. doi: 10.1186/s12881-015-0254-2 (PMC4653905; doi:10.1186/s12881-015-0254-2)

**NPA**

**$\alpha$ -Dbs**

**Tonsil**

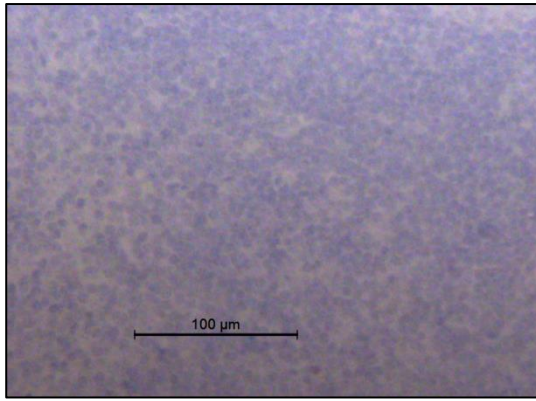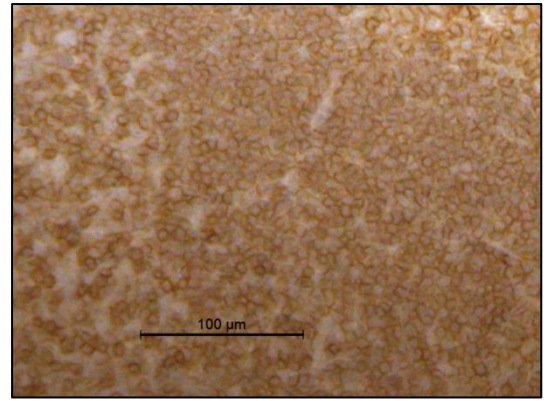

**Synovium**

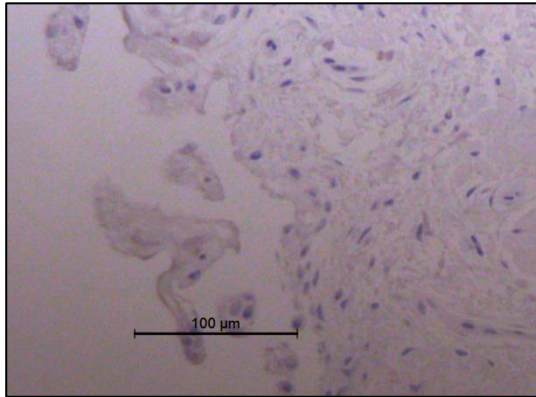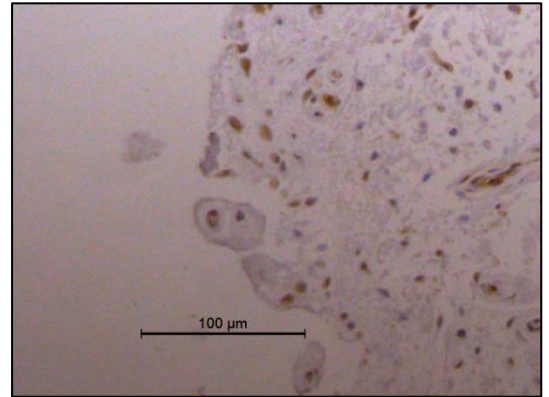

**Fat pad**

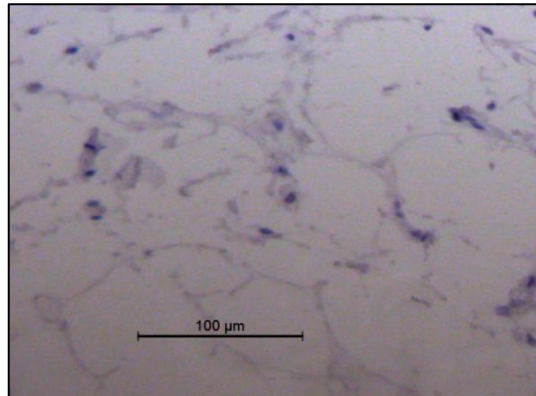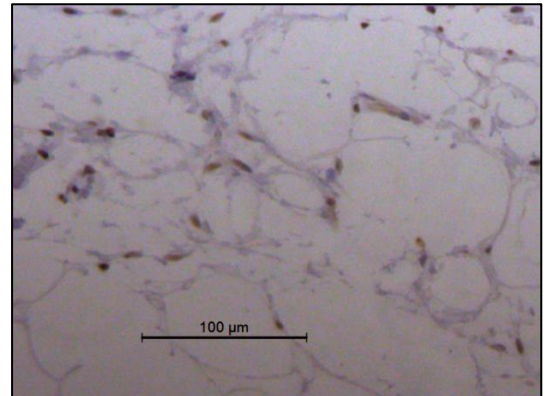

**Cartilage**

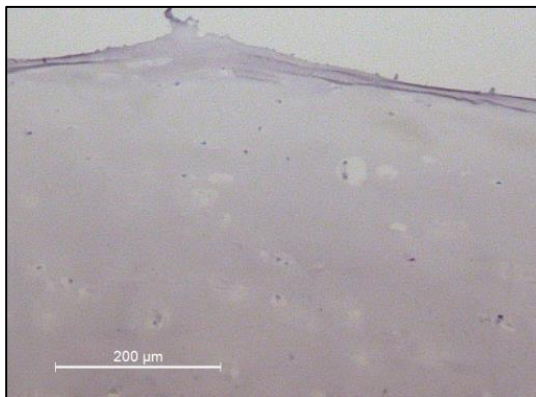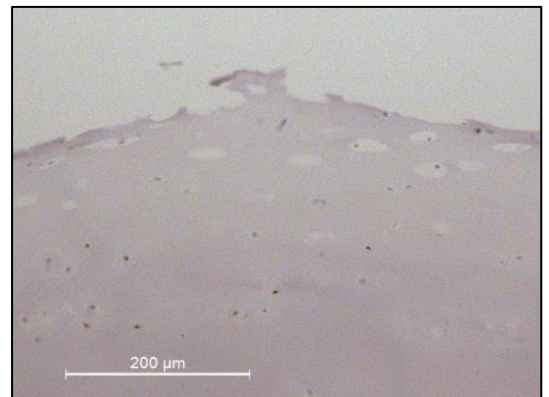

Supplement: Additional file 4: — DBS protein expression in joint tissues from OA patients. Immunohistochemistry staining of fixed and wax embedded synovium, fat pad and cartilage tissue, with healthy tonsil tissue used as a positive control. Tissue was stained with an anti-DBS primary antibody (α-DBS). Also shown is the no primary antibody (NPA) control for each tissue. Bars = 100 μm (tonsil, synovium, fat pad) or 200 μm (cartilage). (PDF 371 kb) [file 12881_2015_254_MOESM4_ESM.pdf]
