# Supplementary material for: Expression analysis of the osteoarthritis genetic susceptibility locus mapping to an intron of the MCF2L gene and marked by the polymorphism rs11842874
Source: BMC Med Genet. 2015 Nov 19;16:108. doi: 10.1186/s12881-015-0254-2 (PMC4653905; doi:10.1186/s12881-015-0254-2)
Supplement: Additional file 5: — An eQTL that regulates MCF2L gene expression in skeletal muscle and oesophagus muscularis. A search of the GTEx website identified an MCF2L eQTL operating in skeletal muscle and oesophagus muscularis. The eQTL is located in a 7 kb LD block containing ten SNPs in high LD (r2 > 0.8). It is approximately 42 kb downstream of the rs11842874 LD block and is in low LD with this block. The table lists the ten SNPs and their r2 and D’ values relative to rs11842874. HG19 coordinates refer to human genome version 19. (PDF 217 kb) [file 12881_2015_254_MOESM5_ESM.pdf]

Addditional file 5. An eQTL that regulates *MCF2L* gene expression in skeletal muscle and oesophagus muscularis. A search of the GTEx website identified an *MCF2L* eQTL operating in skeletal muscle and oesophagus muscularis. The eQTL is located in a 7 kb LD block containing ten SNPs in high LD ($r^2 > 0.8$). It is approximately 42 kb downstream of the rs11842874 LD block and is in low LD with this block. The table lists the ten SNPs and their r^2 and D′ values relative to rs11842874. HG19 coordinates refer to human genome version 19.

			Linkage disequilibrium with rs11842874	
SNP	Distance from rs11842874 (bp)	HG19 coordinates	r^2	D′
rs497724	41224	chr13:113694509	0.1	1
rs527264	42034	chr13:113735733	0.1	1
rs546818	45009	chr13:113739518	0.1	1
rs487612	46769	chr13:113741278	0.1	1
rs522511	42583	chr13:113737092	0.096	1
rs522487	42590	chr13:113737099	0.096	1
rs514181	47342	chr13:113741851	0.096	1
rs498836	42876	chr13:113737385	0.093	1
rs491455	43666	chr13:113738175	0.047	0.639
rs1755693	44294	chr13:113738803	0.047	0.639
