# Supplementary material for: Expression analysis of the osteoarthritis genetic susceptibility locus mapping to an intron of the MCF2L gene and marked by the polymorphism rs11842874
Source: BMC Med Genet. 2015 Nov 19;16:108. doi: 10.1186/s12881-015-0254-2 (PMC4653905; doi:10.1186/s12881-015-0254-2)
Supplement: Additional file 6: — Bioinformatics database search of the SNPs in high LD with rs11842874. Detailed are the chromatin states around the SNPs in several types of joint-related cell from various origins including osteoblasts, mesenchymal stem cells, differentiated chondrocytes and adipocytes; characterising the regulatory properties of the region. (PDF 213 kb) [file 12881_2015_254_MOESM6_ESM.pdf]

Additional File 6. RegulomeDB bioinformatics database search of the SNPs in high LD with rs11842874. Detailed are the chromatin states around the SNPs in several types of joint-related cell from various origins. HG19 coordinates refer to the human genome version 19. Motif specifies any recognised transcription factor binding consensus sequences which incorporate the SNP.

					Chromatin state					
SNP	Distance from rs11842874 (bp)	HG19 coordinates	Motif	Transcription factor binding	Osteoblast primary cells	Adipose derived MSC cultured cells	MSC derived adipocyte cultured cells	MSC derived chondrocyte cultured cells	Bone marrow derived cultured MSC	H1 derived MSCs
rs75351348	8821	chr13:113685688	No data	No data	Weak repressed polycomb	Repressed polycomb	Weak repressed polycomb	Repressed polycomb	Weak repressed polycomb	Weak repressed polycomb
rs118021693	5763	chr13:113688746	No data	No data	No data	No data	No data	No data	No data	No data
rs76623552	2963	chr13:113691546	No data	No data	Weak repressed polycomb	Weak repressed polycomb	Quiescent/low	Weak repressed polycomb	Weak repressed polycomb	Weak repressed polycomb
rs11842874	0	chr13:113694509	No data	No data	Repressed polycomb	Weak repressed polycomb	Weak repressed polycomb	Repressed polycomb	Repressed polycomb	Repressed polycomb
rs1888227	720	chr13:113695229	RREB-1	No data	Repressed polycomb	Enhancers	Enhancers	Weak repressed polycomb	Repressed polycomb	Repressed polycomb
rs113120232	1271	chr13:113695780	Pax-4 Pax-5 SP1	USF1 USF2 MAX	Weak repressed polycomb	Enhancers	Enhancers	Weak repressed polycomb	Weak repressed polycomb	Weak repressed polycomb
rs79866171	2546	chr13:113697055	PPARdirectrepeat1 COUPdirectrepeat1	No data	Weak repressed polycomb	Weak repressed polycomb	Weak repressed polycomb	Repressed polycomb	Repressed polycomb	Repressed polycomb
